# Switching to boosted protease inhibitor plus a second antiretroviral drug (dual therapy) for treatment simplification: a multicenter analysis

**DOI:** 10.7448/IAS.17.4.19802

**Published:** 2014-11-02

**Authors:** Mauro Zaccarelli, Massimiliano Fabbiani, Carmela Pinnetti, Vanni Borghi, Alberto Giannetti, Gaetana Sterrantino, Patrizia Lorenzini, Alessandra Latini, Laura Loiacono, Manuela Colafigli, Adriana Ammassari, Gabriella D'Ettorre, Maddalena Plazzi, Simona Di Giambenedetto, Andrea Antinori

**Affiliations:** 1 Clinical Department, National Institute for the Infectious Diseases “Lazzaro Spallanzani”, Rome, Italy; 2 Department of Infectious Diseases, Catholic University of the Sacred Heart, Rome, Italy; 3 Clinic of Infectious Diseases, Azienda Ospedaliero Universitaria, Modena, Italy; 4 Division of Infectious Diseases, ‘Careggi' Hospital, Florence, Italy; 5 Clinic of Dermatology and Infectious Diseases, “San Gallicano” Institute, Rome, Italy; 6 Clinic of Infectious Diseases, Policlinico Universitario, Rome, Italy

## Abstract

**Background:**

To assess the role of drugs used in dual therapy (DT), as cART simplification, over the risk of treatment failure.

**Materials and Methods:**

Patients starting DT regimen composed by a boosted protease inhibitor (PI/r): darunavir (DRV/r), lopinavir (LPVr) or atazanavir (ATV/r) plus a second drug: raltegravir (RAL), maraviroc (MRV) etravirine (ETR), lamivudine (3TC) or tenofovir (TDF), this one generally used in HBV co-infected patients, were included. The effect of each drug as well as other clinical and virological cofactors over treatment failure was assessed using survival analysis.

**Results:**

Overall, 480 patients from six reference Italian centres were included: all switched to DT with HIV-RNA <500 cp/µL, 376 of them at <50 cp/µL. Patients who switched at <50 cp/µL showed a significant lower risk of treatment failure (13.3% versus 23.3% at 1 year and 28.0% versus 44.6% at 3 years, p=0.005), thus the analysis was focused on this subgroup. Among the patients who switched at <50 cp/µL, the proportion of drug used in DT was: DRV/r 63.0%, RAL 53.7%, ETR 19.4%, ATV/r 18.4%, MRV 17.3%, LPV/r 12.8%, TDF 6.4% and 3TC 5.9%; DRV/r-RAL was the most widely used combination: 32.5%. Treatment failure was observed in 78 patients, of whom 38 virological and 35 for toxicity/intolerance, one patient died during follow-up and four patients interrupted for personal decision with undetectable HIV-RNA. At Cox Model, adjusted by gender, age, non-Italian origin, AIDS diagnosis, time on cART, number of regimens, CD4 nadir, baseline CD4, all the drugs had a positive effect on probability of failure (Figure), however the effect was significant for DRV/r (HR:0.21, 95% CI 0.07–0.59, p=0.03), ATV/r (0.30, 0.09–0.97, p=0.044) and RAL (0.37, 0.15–0.93, p=0.034); higher CD4 count at baseline was also associated with lower risk of failure while number of previous regimens with a higher risk. Moreover, ATV/r was found significant associated with significant higher risk of failure by toxicity (as well as LPV/r) but lower risk of virological failure, while both 3TC and RAL with significant lower risk of toxicity.

**Conclusions:**

Our analysis suggest that using PI/r-based DL is highly effective if switching from HIV-RNA <50 cp/µL; DL should be used with caution in patients with low CD4 count and longer history of treatment; DRV/r is the best compromise among PI/r, ATV/r is effective but is associate with frequent interruption by toxicity; RAL showed high tolerability so that its use is related to the lowest risk of failure as second drug.

**Figure 1:**
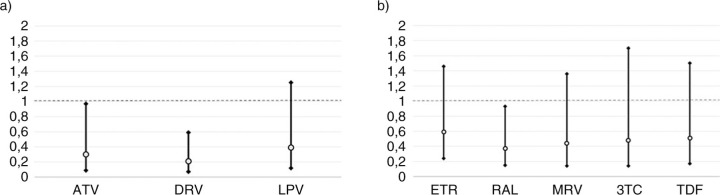
Adjusted hazard ratio for dual-therapy treatment failure (Cox Model): a) boosted PI; b) second drug.

